# Estimation of a preliminary therapeutic reference range for children and adolescents with tic disorders treated with tiapride

**DOI:** 10.1007/s00228-020-03000-0

**Published:** 2020-09-28

**Authors:** Stefanie Fekete, K. Egberts, T. Preissler, C. Wewetzer, C. Mehler-Wex, M. Romanos, M. Gerlach

**Affiliations:** 1 Centre for Mental Health, Department of Child and Adolescent Psychiatry, Psychosomatics and Psychotherapy, University Hospital of Würzburg, Margarete-Höppel-Platz 1, 97080 Würzburg, Germany; 2 Arbeitsgemeinschaft für Neuropsychopharmakologie und Pharmakopsychiatrie (AGNP)-Work group “Kinder- und jugendpsychiatrische Pharmakologie”, 1. Vorsitzender Prof. Dr. med. M. Kölch, Kliniken für Kinder- und Jugendpsychiatrie, Psychotherapie und Psychosomatik, Vivantes Klinikum im Friedrichshain, Vivantes Klinikum Neukölln, Landsberger Allee 49, 10249 Berlin, Germany; 3 Competence Network Therapeutic Drug Monitoring (TDM-KJP e.V.), Centre for Mental Health, Department of Child and Adolescent Psychiatry, Psychosomatics and Psychotherapy, University Hospital of Würzburg, Margarete-Höppel-Platz 1, 97080 Würzburg, Germany; 4 Department of Child and Adolescent Psychiatry and Psychotherapy, Kepler University Clinics GmbH, Linz, Austria; 5 Clinics of the City Cologne GmbH, Clinic for Child and Adolescent Psychiatry and Psychotherapy, Cologne, Germany; 6 Department of Child and Adolescent Psychiatry and Psychotherapy, University Hospital of Ulm, Ulm, Germany; 7 HEMERA Private Hospital for Mental Health, Adolescents and Young Adults, Bad Kissingen, Germany

**Keywords:** Tourette syndrome, Therapeutic drug monitoring, Serum concentration, Paediatrics, Pharmacokinetics

## Abstract

**Purpose:**

Tiapride is commonly used in Europe for the treatment of tics. The aim of this study was to examine the relationship between dose and serum concentrations of tiapride and potential influential pharmacokinetic factors in children and adolescents. In addition, a preliminary therapeutic reference range for children and adolescents with tics treated with tiapride was calculated.

**Methods:**

Children and adolescents treated with tiapride at three university hospitals and two departments of child and adolescents psychiatry in Germany and Austria were included in the study. Patient characteristics, doses, serum concentrations, and therapeutic outcome were assessed during clinical routine care using standardised measures.

**Results:**

In the 49 paediatric patients (83.7% male, mean age = 12.5 years), a positive correlation was found between tiapride dose (median 6.9 mg/kg, range 0.97–19.35) and serum concentration with marked inter-individual variability. The variation in dose explained 57% of the inter-patient variability in tiapride serum concentrations; age, gender, and concomitant medication did not contribute to the variability. The symptoms improved in 83.3% of the patients. 27.1% of the patients had mild or moderate ADRs. No patient suffered from severe ADRs.

**Conclusions:**

This study shows that tiapride treatment was effective and safe in most patients with tics. Compared with the therapeutic concentration range established for adults with Chorea Huntington, our data hinted at a lower lower limit (560 ng/ml) and similar upper limit (2000 ng/ml).

## Introduction

The central acting benzamide tiapride, a selective antagonist of dopamine D2 receptors with weak antipsychotic properties, is used commonly in Europe for the treatment of tics due to the decades of positive clinical experience with this agent [1, 2]. It is also mentioned as a treatment option in patients with tics in the new Practice Guideline of the American Academy of Neurology [3]. Especially the D2 receptors in the striatum are considered to play a central role in the treatment of tic disorders [4]. In Germany, tiapride is one of the ten most commonly prescribed antipsychotic substances in paediatrics [5] and is used as first-line treatment, particularly in Tourette syndrome [2]. However, it is not approved for the treatment of tic disorders [2, 6]. Therapeutic drug monitoring (TDM) is a proactive pharmacovigilance measure used to improve the safety and efficiency of on- and especially off-label psychopharmacological treatment [7]. TDM is based on the hypothesis that brain concentrations of neuro-/psychoactive drugs are related to response and that serum concentrations correlate better with brain concentrations than the prescribed dose [8]. Therefore, serum concentrations should adequately predict the outcome (therapeutic effect and adverse drug reactions (ADRs)) following application of a psychoactive drug [9]. As a therapeutic reference range for tiapride is only available for adult patients with Chorea Huntington [10], the aim of this prospective naturalistic study was to examine the relationship between dose and serum concentrations of tiapride and factors influencing serum concentrations. In addition, a therapeutic reference range for children and adolescents with tics treated with tiapride was estimated.

## Materials and methods

The study was approved by the local ethic committee (study number 27/04) and carried out according to the Declaration of Helsinki. There was no need for written informed consent as the investigation of serum concentration was part of routine clinical treatment.

### Setting and study population

Patient data and blood samples from children and adolescents treated with tiapride tablets were prospectively collected from three university hospitals (Würzburg, Ulm, Erlangen) and two departments of child and adolescent psychiatry (Cologne-Hohlweide, Linz) in Germany and Austria between January 2007 and June 2014. All clinics took part in the routine TDM service of the Center of Mental Health of the University Hospital Würzburg and are members of the competence network for TDM in child and adolescent psychiatry (www.tdm-kjp.com), described in detail elsewhere [11]. All patients with a TDM assessment for the pharmacological treatment with tiapride were included regardless of diagnosis or treatment setting (inpatient, outpatient, day-unit). Patients were excluded from analysis if serum concentrations were not collected in a steady state (24–48 h of constant dose administration), and peak serum concentration (*c*_max_) conditions (2 h after oral administration) or values were below the limit of detection, e.g., in the case of absolute non-compliance. If more than one assessment of tiapride was available for one patient, the last chronological instance was considered for evaluation to avoid the problem of multiple determinations. All available serum concentrations were only included for the intra-individual analyses.

### Assessment of patient characteristics and clinical outcome

Patients’ demographic, psychiatric, and outcome data was assessed in a structured and standardised way. Initially, all patients received a physical, neurological, and psychiatric examination including measurements of vital signs, body length, body weight, electrocardiogram, and laboratory analyses for hepatic and renal function. In addition, the presence of a current infection, caffeine consumption, and smoking habits was assessed. Data was documented in a form that included the following clinical information: date of birth, sex, psychiatric diagnosis, dosage of tiapride, type and dosage of psychiatric co-medications (if any), time and date of blood withdrawal, and date and time of the last dose adjustment.

The severity of psychopathology and changes therein were assessed by the attending physicians at the time of the blood withdrawal using the clinical global impression (CGI) scale, the subscale for severity of illness (CGI-S), and the CGI subscale for global improvement (CGI-I) [12]. When applying the CGI-I scale, improvement must be compared with the original drug-naive state of symptoms. According to the CGI-I manual, the following categories were used: 1 = very much improved; 2 = much improved; 3 = moderately improved; 4 = no change; 5 = minimally worse; and 6 = much worse. ADRs were assessed with the side effect rating scale from the Udvalg for Kliniske Undersogelser (UKU) using the following categorisation: 0 = no ADRs, 1 = mild, 2 = moderate, and 3 = severe ADRs [13]. The UKU scale consists of 12 items (e.g., sedation, polydipsia). On the request form, further side effects could be documented under “other ADRS”.

### Measurement of tiapride serum concentration

Tiapride serum concentrations were determined in the TDM laboratory of the Centre of Mental Health at the University Hospital of Würzburg. Blood was collected from cubital veins in 7.5-ml monovettes without coagulants or additives. Collection took place during steady-state *c*_max_ conditions (2 hours) because tiapride has an elimination half-life about 4 h [10, 14].

The blood was centrifuged at 3000 rpm (1851*g*) for 10 min and, for samples from Würzburg, analysed immediately. Samples sent to Würzburg were centrifuged and analysed within 3 days after postage to the TDM laboratory. If the samples were not analysed within 5 days, they were frozen at − 20 °C (max. 3 months).

Serum concentrations of tiapride were analysed with an automated column-switching method coupled with an isocratic high-performance liquid chromatography system and a variable ultraviolet detector (Agilent LC Systems, Series 1100; Agilent Technologies Inc., Santa Clara) as has been described for other psychotropic drugs in detail elsewhere [15]. Wavelength for UV detection was set at 229 nm. The absolute extraction recovery for tiapride was 89 %. The intra-assay was 0.86%, and inter-assay coefficient of variation was 2.47%. The method was linear in a range of 2–6750 ng/ml (*r* = 0.99), and the lower limit of detection was 2 ng/ml.

Chemicals and solvents with appropriate levels of purity as well as tiapride for calibration and controls were purchased commercially from Sigma-Aldrich in Munich, Germany.

### Estimation of a preliminary therapeutic reference range of tiapride in children and adolescents with tic disorders

According to the consensus guidelines for therapeutic drug monitoring in neuropsychopharmacology [9], a preliminary therapeutic reference range should be determined using the arithmetic mean ± SD range of drug concentrations in the blood of patients who responded to the medication. For calculation, only values obtained from patients treated for tic disorders were used.

### Data analysis

Before analysis, all data was anonymised. Statistical analyses were performed with the software SPSS, version 26. All values are presented as mean ± SD, median and range. The Kolmogorov-Smirnov test was applied to evaluate variables for Gaussian distribution.

To evaluate the relationship between tiapride dose, serum concentration, influencing factors (e.g., co-medication), and clinical outcome, Pearson correlation coefficients and multivariate linear regression were used. Group differences were assessed by independent *t* test, Man-Whitney *U* test, and one-way analysis of variance (ANOVA). Statistical significance was defined as *p* < 0.05.

## Results

### Study population

Data from 49 paediatric patients (41 male) treated with tiapride was analysed. The patients’ characteristics are summarised in Table 1. The mean age was 12.5 years (SD 2.8, range 7.0–18.4 years, median 12.5). More than half of the patients were children under the age of 14. The vast majority of patients received tiapride to treat tic disorders (ICD-10 F95.) and in particular Tourette syndrome. 55.1 % of the patients were administered tiapride combined with one or more concomitant psychotropic or somatic drug; 70.3 % of these patients received tiapride plus one concomitant medication; 29.7 % received two or three additional drugs. Most of the patients were classified as “markedly ill” (CGI-S: mean = 4.7; SD = .93; median = 5).

**Table 1:** Characteristics of the patients treated with tiapride (*N* = 49)

Clinical center, *N* (%)	
Wuerzburg, Germany	22 (44.9)
Linz, Austria	12 (24.5)
Cologne-Hohlweide, Germany	7 (14.3)
Ulm, Germany	7 (14.3)
Erlangen, Germany	1 (2.0)
Setting, *N* (%)	
Outpatient care	16 (32.7)
Day unit	6 (12.2)
Inpatients	24 (49.0)
Information not given	3 (6.1)
Sex, *N* (%)	
Male	41 (83.7)
Female	8 (16.3)
Age (years), mean ± SD	12.5 ± 2.8 (range 7.0–18.4)
Children ≤ 14 years, *N* (%)	31 (63.3)
Adolescents > 14 years, *N* (%)	18 (36.7)
Weight (kg), mean ± SD	47.4 ± 15.3 (range 22.0–85.3)
Height (cm), mean ± SD	153.6 ± 16.3 (range 115–188)
Body mass index (kg/m^2)^, mean ± SD	19.5 ± 3.6 (range 14.6–29.0)
Most common ICD diagnoses, *N* (%)	
Tic disorder (F95.)	44 (91.7)
Chronic motor or vocal tic disorder (F95.1)	5 (10.4)
Tourette syndrome (F95.2)	30 (62.5)
Tic disorder, unspecified (F95.9)	9 (18.8)
Other ICD diagnoses, *N* (%)	4 (8.3)
Attention-deficit hyperactivity disorder, predominantly hyperactive type (F90.1)	2 (4.2)
Atypical anorexia nervosa (F50.01)	1 (2.1)
Pervasive developmental disorders (F84.4)	1 (2.1)
Tiapride monotherapy/psychiatric co-medication, *N* (%)	22/27 (44.9/55.1)
Psychostimulants	
Methylphenidate	12 (33.3)
Amphetamine	4 (11.1)
Non-Stimulant ADHD medication	
Atomoxetine	4 (11.1)
Antipsychotics	
Chlorprothixene	2 (5.6)
Aripiprazole	2 (5.6)
Risperidone	2 (5.6)
Quetiapine	1 (2,78)
Antidepressants	
Sertraline	3 (8.3)
Fluoxetine	1 (2.8)
Fluvoxamine	1 (2.8)
Tranquiliser	
Lorazepam	2 (5.6)
Somatic co-medication	
Clindamycin	1 (2.8)
Dronabinol	1 (2.8)

Mean daily dose of tiapride was 354 mg (SD 216, range 50–900, median 300). More than half of the patients (55.8%) received the daily dose as a single dose. About one-third of the patients received tiapride three times,, and one in ten received a dose twice a day. The mean body weight-adjusted dose was 7.7 mg/kg (SD 4.6, range 1.0–19.4, median 6.9). There are no differences in the prescribed daily dose of tiapride between children < 14 years and adolescents (*t* = .05, *p* = .96), boys and girls (*U* = 142.00, *p* = .57), or patients with tiapride monotherapy and those with concomitant medications (*U* = 353.00, *p* = .26) (see Table 2).

**Table 2:** Correlation of dose and serum concentration of tiapride in different populations (mean, ± SD, median)

Patients (*N*)	Tiapride dose (mg/day), mean ± SD median	Serum concentration (ng/ml)	Correlation (*r*_*s*_)	Significance (*p*)
All (49)	354 ± 216 300	1324 ± 804 1193	.78	< 0.001*
Male (41)	362 ± 218 300	1299 ± 711 1153	.80	< 0.001*
Female (8)	313 ± 213 275	1452 ± 1232 1247	.80	= 0.017*
Children < 14 years (31)	352 ± 198 300	1308 ± 766 1221	.73	< 0.001*
Adolescents ≥ 14 years (18)	356 ± 250 300	1353 ± 887 1077	.81	< 0.001*
Monotherapy (22)	307 ± 176 300	1190 ± 785 1132	.70	< 0.001*
Co-medication (27)	392 ± 240 300	1434 ± 817 1270	.80	< 0.001*
Tic disorders (44)	379 ± 212 300	1379 ± 800 1218	.74	< 0.001*
Other disorders (4)	130 ± 76 100	685 ± 556 546		

### Tiapride serum concentrations in relation to tiapride doses

**Fig. 1:**
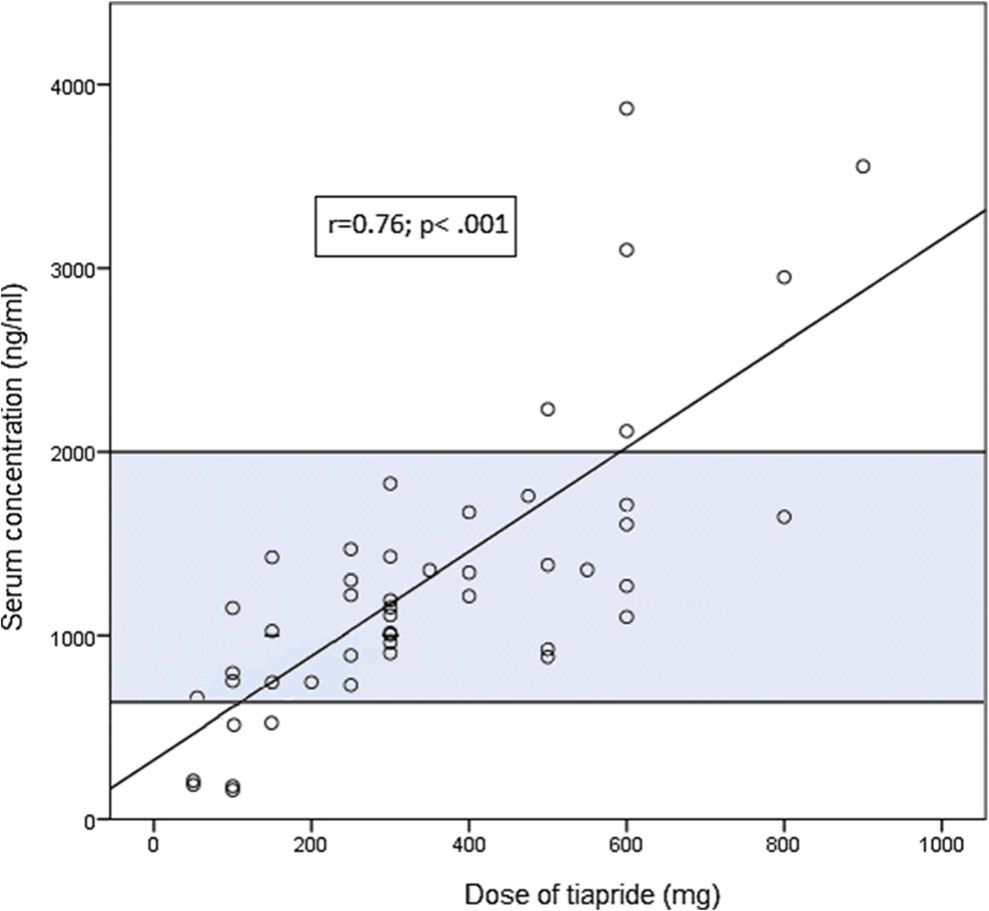
Relationship between dose and serum concentration of tiapride. The preliminary therapeutic reference range of tiapride in children and adolescents with tic disorders is highlighted

The mean tiapride serum concentration (*N* = 49) was 1324 ng/ml (SD 804, range 156–3869, median 1193). As shown in Fig. 1, there was a positive medium linear relationship between dose of tiapride and the serum concentration (*r* = .76, *p* < .001). Variation in dose was responsible for 57% of the variability in serum concentrations (*R* = .76; *R*^2^ = .58) in the total sample. Table 2 shows the relationships between the prescribed doses and the measured serum concentrations in the different subgroups of the study population. There was no difference in serum concentrations between boys and girls (t = − .49; *p* = .63), children and adolescents (*U* = 267.00; *p* = .80), BMI (*U* = 90.00; *p* = .73), or patients receiving co-medication and tiapride as monotherapy (*U* = 365.00; *p* = .17).

The mean dose-corrected serum concentration (C/D) was 4.4 (ng/ml)/(mg/day) (SD 2.4, range 1.6–13.1, median 3.7). There was an intra-individual difference (SD 1.60 (ng/ml)/(mg/day)) between C/D of the first and second measurement (*t* = − .23, *p* = .83) in patients with more than one available measurement (*N* = 14). The inter-individual variability of tiapride serum concentrations was (SD 2.36 (ng/ml)/(mg/day)) (*N* = 49).

### Clinical outcome during treatment with tiapride

The symptoms of tics improved by varying degrees in 83.3% of the patients with a mean CGI-I of 1.25 (SD .84, median 1); for 13 patients, the information about the treatment effect was missing. According to CGI-I, the symptoms were rated “moderately better” in 41.7 % and “much better” in 38.9 %. One patient benefited “very much” from treatment with tiapride (2.8%). Only the minority of patients (16.7 %) was non-responders as their symptoms were rated as “unchanged” (13.9%) or “minimally worse” (2.8 %). Mean serum concentration of patients responding moderately, much, and very much improved (*N* = 30) was 1411 ng/ml (SD 867 ng/ml, range 156–3869 ng/ml, median 1218).

For most of the patients in the study population (72.9%), no ADRs were documented at all (UKU = 0), and severe ADRs did not occur in any patient. One or more “mild” or “moderate” ADRs (UKU ≥ 1) were reported in 13 patients (*N* = 18). Concomitant medication did not affect the incidence of ADRs (*t* = .20; *p* = .84).

The most commonly reported ADR was fatigue (*N* = 6). ADRs that were not recorded on the UKU scale but documented on the request form under “other ADRs” included an increase in prolactin (*N* = 1) and weight gain (*N* = 4). By using the recommended method of the consensus guidelines, the preliminary therapeutic reference range of children and adolescents with tic disorders (*N* = 28) was determined between 560 and 2000 ng/ml.

## Discussion

In this naturalistic study of children and adolescents treated with tiapride, we found a positive linear relationship between the dose and *c*_max_ of tiapride. The variation in dose explained 57% of the inter-patient variability in tiapride serum concentrations. Age, gender, and concomitant medication did not contribute to the variability in serum concentrations of tiapride. Tiapride treatment in patients with tics was documented as effective in nearly 85% of cases. Using the arithmetic mean ± SD range of drug concentrations in the blood of responders to the medication, we calculated a preliminary therapeutic reference range of 560–2000 ng/ml.

### Study population

In our study, boys were overrepresented. This is in line with the distribution of tic disorders (three to four times more boys are affected) [16]. Tics increase in severity to a climax around the age of 10 to 12 years, which is consistent with mean age in our study (mean age 12.5 years, SD 2.8). A high rate of concomitant psychotropic drugs such as methylphenidate was reported in our study, which is explained by the high comorbidity of ADHD with tic disorders [17]. The mean daily dose of tiapride (354 mg/day, SD 216) was higher than the dose recommended by Roessner and Rothenberger to treat tic disorders (150–300 mg/day) [1].

### Tiapride serum concentrations in relation to tiapride dose

Consistent with a TDM study of adult patients with tardive dyskinesia [18] we find a positive correlation between tiapride dose and serum concentration (Fig. 1). Tiapride is eliminated almost un-metabolised through renal excretion [14, 19]. Therefore, renal insufficiency may lead to higher serum concentrations of tiapride [19]. Indeed, we observed a *c*_max_ of 3869 ng/ml in a 10-year-old female patient suffering from comorbid chronic renal insufficiency. This is almost three times as high as the mean serum concentration in the total population. Age-specific differences in kidney function might also influence serum concentrations. Renal clearance of tiapride is related to creatinine clearance, which gradually decreases with age [20]. This could explain why a dose of 100 mg in adults showed mean serum concentrations of 1470 ng/ml [18], whereas only a mean of 730 ng/ml was measured in minors treated with 100 mg tiapride in our study.

Boys and girls showed no differences in mean serum concentrations, consistent with data of two pharmacokinetic studies in adult patients with Chorea Huntington and healthy volunteers [10, 14]. Co-medication did not influence the concentration of tiapride, a finding in line with the known pharmacokinetics of tiapride [10, 14].

The high inter-individual variability of dose-related serum concentrations is in accordance with a study in adult patients with tardive dyskinesia [18] but seems to be particularly pronounced in minors [19, 20]. This variability underlines the need for a specific TDM recommendation for children and adolescents. The high variation in serum concentration demonstrates the high inter-patient variability in pharmacokinetics. Differences in the gastrointestinal absorption of tiapride are one possible reason for the observed intra- and inter-individual variability of serum concentration.

In order to be able to recognise this specific pharmacokinetic property as early as possible and thereby be able to make a decision about continuous dosages, a determination of serum concentration in the early phase of therapy would be helpful. Further measurements showed that the initial level is a useful guiding principle for individual dose adjustment, since the intra-individual variability of the dose-related serum concentrations was relatively low during the course of therapy.

### Clinical outcome during treatment with tiapride

In our study, tiapride appears to be effective in children and adolescents with tic disorders and safe with predominantly mild ADRs. This is consistent with a placebo-controlled study with 17 children and adolescents with Tourette syndrome [21]. In our study, the daily dose was slightly higher than in the study done by Eggers et al. [21] (7.7 vs 5.6 mg/kg/day), however, within the recommended daily dosage per kilogramme body weight in children and adolescents with tic disorders (2-10 mg/kg) [2]. No severe ADRs at all were documented in our study. Furthermore, no ADRs were reported in the 10-year-old patient with comorbid chronic renal insufficiency and a serum concentration of 3869 ng/ml.

We found no influence of co-medication on the occurrence of ADRs. In the literature, pharmacodynamic interactions have been reported in the treatment with tiapride and co-medication with antipsychotics such as aripiprazole, risperidone, and quetiapine [22]. These co-medications might increase the risk of QTc prolongation and torsades de pointes arrhythmia [22]. In our study, the above-mentioned concomitant medications did not lead to cardiovascular ADRs in any of the cases.

Two patients showed mild symptoms of extrapyramidal motor ADRs. One of the patients had a serum concentration of 2113 ng/ml (daily dose 600 mg/day, C/D 3.52 (ng/ml)/(mg/day); the other patient showed a high C/D of 7.51 (ng/ml)/(mg/d) during titration phase (daily dose 100 mg, serum concentration 751 ng/ml. In particular, extrapyramidal motor symptoms are a predictor of over dosage or over occupancy of dopaminergic D_2_ receptors in the striatum (> 80% in positron emission tomography (PET) studies) [23]. In the case of tiapride in PET studies with healthy adult volunteers, no receptor occupancy above 80% was observed at a high dose of 600 mg/day [24]. In our study, the mean daily dose of tiapride was 354 mg (SD 216), well below 600 mg/day. This likely explains the very low rate of extrapyramidal motor ADRs and the generally good tolerance of tiapride.

### Estimation of a preliminary therapeutic reference range of tiapride in children and adolescents with tic disorders

The therapeutic reference range is an essential target range for TDM-guided pharmacotherapy. Its estimation includes the determination of a lower and upper limit of therapeutically effective and tolerable drug concentrations in the blood [9]. So far, a generally accepted method for estimation of therapeutic reference range does not exist. In correlating serum concentrations and therapeutic outcomes in clinical trials to determine the therapeutic reference range, methodological restrictions such as placebo response or treatment resistance must be considered [9]. If clinical outcome and ADRs correlated with serum concentrations, the receiver operating characteristic (ROC) analysis would be applied for the calculation of the lower and upper limit of the therapeutic range. Additionally, PET studies could be used to define a therapeutic reference range for antipsychotics by correlating D2 receptor occupancy with serum concentrations in adults. However, PET studies in minors cannot be carried out because of ethical concerns. Therefore, we used the arithmetic mean ± SD range of drug concentrations in blood of responders to the medication to determine a preliminary therapeutic reference range as recommended by the consensus guidelines of TDM in neuropsychopharmacology [9].

## Limitations

The results of our study must be considered in the context of the typical limitations of a naturalistic study. This naturalistic study relies on relatively uncontrolled conditions in a routine clinical treatment with many uncontrolled factors, such as different length of treatment time, different dose intervals, possible deviations in time of blood withdrawal, treatment with co-medication, and unblinded evaluation of the drug effectiveness by the primary caregivers.

A further limitation is the blood collection time, namely, *c*_max_, because deviations from the correct sampling time lead to a higher variability in the measured drug concentrations. For TDM, usually trough levels at steady state are recommended because deviations from the correct sampling time immediately prior to the next dose are less critical for trough samples than during other phases after dose application, since the concentration time curve is relatively flat towards the end of the dosing interval [9]. However, for drugs with a short elimination half-life, such as tiapride, serum concentrations are determined at *c*_max,ss_ because of analytical considerations and clinical effects that correlate with *c*_max,ss_. To more precisely interpret the pharmacokinetic influencing factors of tiapride, further information is needed, like kidney function (creatinine, estimated glomerular filtration rate).

Data obtained in a routine TDM service like ours are only limitedly suitable for scientifically validating the relationship between serum concentration and treatment outcome (treatment response and ADRs) of psychotropic drugs. Determining TDM guidelines for tiapride is especially difficult because natural fluctuations underlie tics (see [17]). The selection of the chronologically last available valid TDM assessment per patient for the analysis in our study does not allow for the assessment of treatment effects in the titration phase, where the best quantifiable response could be expected. Instead, this data reflects effects at different times in the course of (long-term) therapy. A more specific study would attempt to correlate clinical outcome parameters with inter-individual serum concentrations over a longer period with more measurements per patient. All in all, it is difficult to demonstrate if drug concentrations correlate with therapeutic outcomes in flexible dose studies like ours. This would require a more controlled study design, larger numbers of patients, fixed dose regimens, and the use of specific psychometrical tools for assessing symptom improvement (e.g. the Yale Global Tic Severity Scale [25] and Paediatric Adverse Event Rating Scale (PAERS)) as well as standardised TDM measurement times.

## Conclusion

This naturalistic study shows that tiapride treatment is effective and safe in most children and adolescents with tics disorders. A positive correlation between dose and serum concentrations of tiapride with a high inter-individual and relatively low intra-individual variability in serum concentrations was demonstrated. The high inter-individual variation underlines the need for a specific TDM recommendation. Compared with the therapeutic concentration range established for adults with Chorea Huntington, our data hinted at a lower lower limit (560 ng/ml)) and similar upper limit (2000 ng/ml). Taking the limitations of the present naturalistic study into account, our results should be validated in larger samples and studies with more controlled designs.
