# Correction to: Estimation of a preliminary therapeutic reference range for children and adolescents with tic disorders treated with tiapride

**DOI:** 10.1007/s00228-021-03159-0

**Published:** 2021-05-28

**Authors:** Stefanie Fekete, K. Egberts, T. Preissler, C. Wewetzer, C. Mehler-Wex, M. Romanos, M. Gerlach

**Affiliations:** 1 Department of Child and Adolescent Psychiatry, Psychosomatics and Psychotherapy, Centre for Mental Health, University Hospital of Würzburg, Margarete-Höppel-Platz 1, 97080 Würzburg, Germany; 2 Arbeitsgemeinschaft Für Neuropsychopharmakologie Und Pharmakopsychiatrie (AGNP)-Work Group “Kinder- Und Jugendpsychiatrische Pharmakologie”, Psychotherapie Und Psychosomatik, Kliniken Für Kinder- Und Jugendpsychiatrie, Vivantes Klinikum Im Friedrichshain, 1. Vorsitzender Prof. Dr. med. M. KölchVivantes Klinikum Neukölln, Landsberger Allee 49, 10249 Berlin, Germany; 3 Competence Network Therapeutic Drug Monitoring (TDM-KJP E.V.), Department of Child and Adolescent Psychiatry, Psychosomatics and Psychotherapy, Centre forMental Health, University Hospital ofWürzburg, Margarete-Höppel-Platz 1, 97080 Würzburg, Germany; 4 Department of Child and Adolescent Psychiatry and Psychotherapy, Kepler University Clinics GmbH, Linz, Austria; 5 Clinic for Child and Adolescent Psychiatry and Psychotherapy, Clinics of the City Cologne GmbH, Cologne, Germany; 6 Department of Child and Adolescent Psychiatry and Psychotherapy, University Hospital of Ulm, Ulm, Germany; 7 HEMERA Private Hospital for Mental Health, Adolescents and Young Adults, Bad Kissingen, Germany

## Correction to: European Journal of Clinical Pharmacology (2021) 77:163–170 https://doi.org/10.1007/s00228-020-03000-0

The article Estimation of a preliminary therapeutic reference range for children and adolescents with tic disorders treated with tiapride, written by Stefanie Fekete, K. Egberts, T. Preissler, C. Wewetzer, C. Mehler-Wex, M. Romanos and M. Gerlach, was originally published electronically on the publisher’s internet portal on 28 September 2020 without open access. With the author(s)’ decision to opt for Open Choice the copyright of the article changed on 11 May 2021 to © The Author(s) 2021 and the article is forthwith distributed under a Creative Commons Attribution 4.0 International License, which permits use, sharing, adaptation, distribution and reproduction in any medium or format, as long as you give appropriate credit to the original author(s) and the source, provide a link to the Creative Commons licence, and indicate if changes were made. The images or other third party material in this article are included in the article’s Creative Commons licence, unless indicated otherwise in a credit line to the material. If material is not included in the article’s Creative Commons licence and your intended use is not permitted by statutory regulation or exceeds the permitted use, you will need to obtain permission directly from the copyright holder. To view a copy of this licence, visit http://creativecommons.org/licenses/by/4.0.

